# Electrocatalytic Volleyball: Rapid Nanoconfined Nicotinamide Cycling for Organic Synthesis in Electrode Pores

**DOI:** 10.1002/anie.201814370

**Published:** 2019-02-14

**Authors:** Clare F. Megarity, Bhavin Siritanaratkul, Rachel S. Heath, Lei Wan, Giorgio Morello, Sarah R. FitzPatrick, Rosalind L. Booth, Adam J. Sills, Alexander W. Robertson, Jamie H. Warner, Nicholas J. Turner, Fraser A. Armstrong

**Affiliations:** ^1^ Department of Chemistry University of Oxford South Parks Road Oxford OX1 3QR UK; ^2^ Manchester Institute of Biotechnology School of Chemistry University of Manchester Manchester M1 7DN UK; ^3^ Department of Materials University of Oxford Parks Road Oxford OX1 3PH UK

**Keywords:** cofactor recycling, electrocatalysis, ferredoxin NADP^+^ reductase, nanoconfinement, nicotinamide

## Abstract

In living cells, redox chains rely on nanoconfinement using tiny enclosures, such as the mitochondrial matrix or chloroplast stroma, to concentrate enzymes and limit distances that nicotinamide cofactors and other metabolites must diffuse. In a chemical analogue exploiting this principle, nicotinamide adenine dinucleotide phosphate (NADPH) and NADP^+^ are cycled rapidly between ferredoxin–NADP^+^ reductase and a second enzyme—the pairs being juxtaposed within the 5–100 nm scale pores of an indium tin oxide electrode. The resulting electrode material, denoted (FNR+E2)@ITO/support, can drive and exploit a potentially large number of enzyme‐catalysed reactions.

Nature has evolved efficient systems whereby coupled enzyme reactions occur in irregular nanoconfined three‐dimensional zones—mitochondria and chloroplasts being prime examples.^1^ In photosynthesis, sunlight is used to regenerate nicotinamide adenine dinucleotide phosphate (NADPH) and adenosine triphosphate (ATP).^2^ Ferredoxin (Fd) transfers two electrons, one at a time, from photosystem I to ferredoxin NADP^+^ reductase (FNR), which is a flavoenzyme that catalyses the conversion of NADP^+^ to NADPH.^2^, ^3^ Fast and efficient recycling occurs within <100 nm in the chloroplast stroma.^2^, ^3^

We discovered recently that FNR binds tightly in the pores of an indium tin oxide (ITO) electrode, where it exhibits rapid electron transfer, reversible electrocatalysis of NADP^+^/NADPH interconversion, and catalytic coupling to a dehydrogenase introduced to the solution.^4^ The electrode was constructed by electrophoretic deposition of ITO particles (diameter <50 nm; Supporting Information, Figure S1) onto a conductive support (Figure 1 A; Figure S1). The interparticle spaces form disordered pores with diameters ranging from 5–100 nm (Figure 1 A; Figure S1), which is similar to chloroplast stroma.^2^, ^3^ A pristine ITO glass electrode (that is, without an electrophoretically deposited porous ITO layer) showed no discernible FNR binding or electrocatalytic activity.

**Figure 1:**
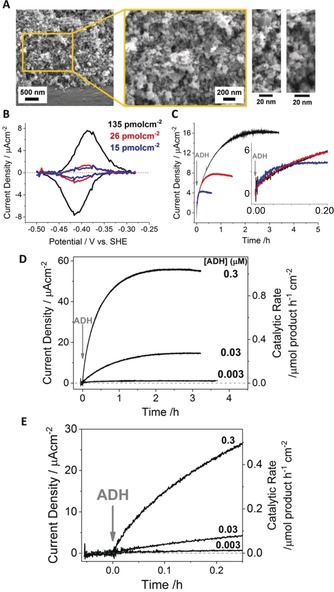
The porous ITO layer and development of catalytic activity when ADH is introduced to a FNR@ITO/graphite electrode A) Cross‐section scanning electron microscopy (SEM) images of the ITO layer. B) Background‐subtracted non‐turnover peaks for FNR (see text). C) Chronoamperograms showing the rate of development of catalytic activity upon introduction of ADH (final concentration of 0.03 μm.) to the three FNR‐preloaded electrodes shown in (B); the insert in (C) is a close‐up of the initial stage. D) Effect of ADH concentration on the rate of rise of catalytic activity for FNR@ITO/graphite electrodes preloaded with similar amounts of FNR (98, 95, and 110 pmol cm^−2^ for 0.3, 0.03, and 0.003 μm ADH experiments, respectively). ADH injected at *t*=0. E) Magnification of the initial 15 min in panel (D). Conditions (C–E): NADPH (5 μm) and 4‐phenyl‐2‐butanol (20 mm) present from the start, electrode held at +0.08 V vs. SHE, 1000 rpm rotation, 20 °C, 2‐(*N*‐morpholino)ethanesulfonic acid (MES, 50 mm), *N*‐[tris(hydroxymethyl)methyl]‐3‐aminopropanesulfonic acid (TAPS, 50 mm), pH 8, cell volume 3 mL.

We now report a massive nanoconfinement effect for two‐enzyme electrocatalysis utilising NADP(H) recycling and show that the fundamental principle enabling enzyme cascades to operate so efficiently in living cells extends to the development of electrodes for controllable and selective organic synthesis. We demonstrate that electrochemically driven cofactor recycling occurs exclusively between FNR and a dehydrogenase enzyme (E2) that is co‐entrapped in the pore, leading also to local enhancement of NADP(H) concentration. The resulting material, denoted “(FNR+E2)@ITO/support”, is highly electrocatalytic because of the combined action of two enzymes.

An alcohol dehydrogenase (ADH, W110A variant)^5^ was adopted as the exemplar enzyme in initial studies, which were extended to include a reductive aminase (RedAm),^6^ an imine reductase ((*S*)‐IRED),^7^ and malic enzyme (ME^8^) catalysing reductive incorporation of CO_2_. Reactions are shown in the proceeding text and some enzyme properties are given in Table S1 (Supporting Information).


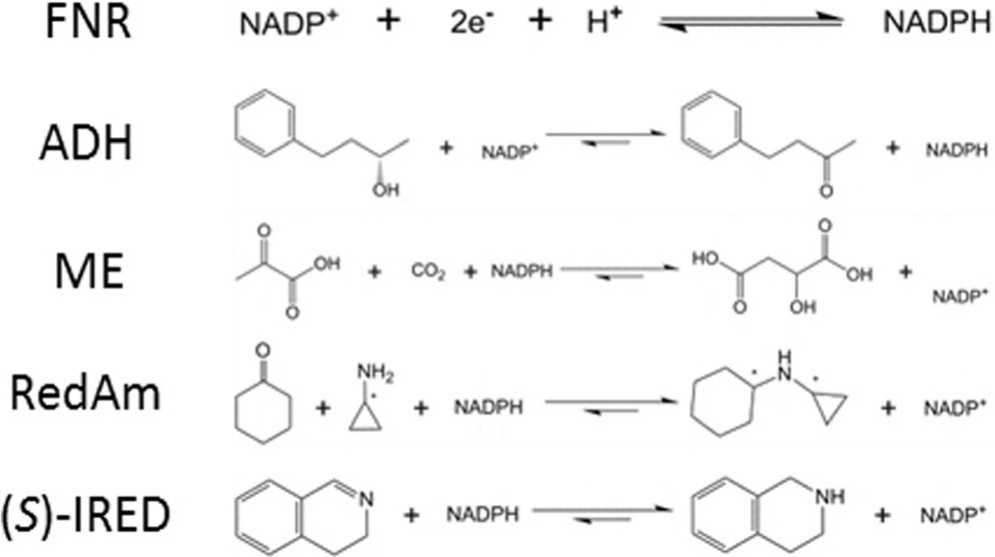


The tight binding of FNR in the ITO pores is quantified by analysing the stable “non‐turnover” cyclic voltammetry peaks measured in the absence of NADP(H), which correspond to rapid, reversible 2‐electron reduction and oxidation of the enzyme's flavin cofactor, FAD (Figure 1 B).4a Different amounts of FNR were pre‐adsorbed at an ITO electrode by placing it in a stirred FNR solution (2.5 μm in buffer, pH 8.0) for various durations. Electroactive coverages were obtained from charges given by the peak areas (Figure 1 B). As a guide, and based on globular diameter, a single packed monolayer of FNR on a flat surface would give a coverage of approximately 5 pmol cm^−2^. In contrast, actual values ranged from 15 to 135 pmol cm^−2^, depending on exposure time.

Each electrode was used to carry out the oxidation of (*S*)‐(+)‐4‐phenyl‐2‐butanol (hereafter, 4‐phenyl‐2‐butanol) catalysed by ADH and monitored by chronoamperometry at +0.08 V versus standard hydrogen electrode (SHE; Figure 1 C). We measured oxidation rather than reduction to facilitate experiments that otherwise require rigorous anaerobicity to avoid current spikes from trace O_2_ when reagents are injected. Upon addition of ADH (final concentration 0.03 μm) to the cell solution (3 mL), already containing NADPH (5 μm), the current (which is directly proportional to catalytic rate; that is, current/2*F*, where *F* is Faraday's constant) rose gradually from zero to reach a limiting value. The right axis here and elsewhere shows the catalytic rate derived from the current using Faraday's constant. Importantly, the curve contains information on two different rates: 1) the slope during the approach to the limiting value represents the rate of development of catalytic activity (that is, how fast the operational electrocatalyst is assembled); 2) the limiting value represents the optimal steady‐state rate of catalytic conversion (moles product/time) that is achieved. A current density of 54 μA cm^−2^ (geometric electrode surface area) corresponds to a catalytic conversion rate of 1 μmol product cm^−2^ h^−1^.

We interpret the development of catalytic activity as being a consequence of the second enzyme (E2) entering the ITO pores and binding close to FNR, and the complementary enzyme partners executing nanoconfined cofactor recycling with a massively enhanced catalytic rate. Production of 4‐phenyl‐2‐butanone was confirmed by ^1^H NMR spectroscopy (Figure S2). The essentially exponential current growth suggests a first‐order process dependent on the number of adsorption sites available to incoming ADH molecules. The electrode with the lowest FNR coverage gave the highest initial rate of ADH adsorption but attained the lowest maximum level. Thus, lower amounts of pre‐adsorbed FNR limit the final catalytic current (activity) but present less resistance to incoming ADH molecules. Figure 1 D presents experiments in which the quantity of pre‐adsorbed FNR was held roughly constant, and three different levels of ADH were introduced. From the magnified view shown in Figure 1 E, it is clear that introducing ADH to the cell does not cause an immediate increase in current, as would be expected were ADH to contribute to the catalytic activity while in solution. The maximum current and rate of rise both increase with ADH concentration in a non‐linear manner (a 10‐fold increase yielding less than a fivefold increase in maximum current). The order of addition was then reversed; that is, FNR was introduced to ITO that had been pre‐exposed to ADH. Unlike FNR, ADH is not an electron‐transfer enzyme, so we could not quantify its adsorption by cyclic voltammetry. Instead, increasing amounts of ADH were preloaded at ITO/graphite by varying the incubation time between 0.3 and 150 min. Each electrode was then rinsed thoroughly before placing it in a cell solution containing substrate and NADPH (5 μm). Upon injecting FNR (final concentration 1.3 μm), the current increased exponentially from zero (Figure 2 A), as observed when ADH was the incoming enzyme. The result indicated that ADH, like FNR, binds strongly to the electrode.

**Figure 2:**
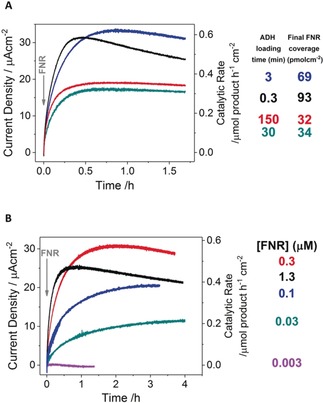
Chronoamperometry experiments showing the development of catalytic activity when FNR is introduced to ITO/graphite electrodes preloaded with ADH. A) FNR (final cell concentration of 1.3 μm) was introduced to ITO electrodes preloaded with different amounts of ADH (achieved by loading ADH (93 μm, 10 μL) for the times shown); the final FNR coverage is listed. B) The FNR concentration was varied while the amount of preloaded ADH was held as constant as possible by dropcasting ADH (93 μm, 5 μL) for 30 min, before thorough rinsing in a stream of pure water. FNR was injected at *t*=0. NADPH (5 μm) and 4‐phenyl‐2‐butanol (20 mm) were present from the start. Conditions: electrode held at +0.08 V vs. SHE, 1000 rpm rotation, 20 °C, MES (50 mm), TAPS (50 mm), pH 8, cell volume 3 mL.

The rate of increase was greatest for the experiment in which ADH had been exposed to ITO for the shortest time (that is, 0.3 min), suggesting that FNR adsorbs more rapidly if less ADH is already present in the pores. Long ADH preloading times gave lower maximum current but higher stability. After each experiment, the electrode was rinsed and placed in a fresh solution devoid of substrates. Cyclic voltammetry verified that the amount of adsorbed FNR increases with decreasing ADH pre‐adsorption.

Figure 2 B presents studies in which the FNR concentration was varied and the preloaded ADH level was kept as uniform as possible by dropcasting for 30 min in each case. The maximum current and rate of binding of FNR both increase non‐linearly with FNR concentration between 0.03 and 0.3 μm. The current for 0.003 μm FNR was barely visible, while 1.3 μm FNR yielded the most rapid increase but gave the greatest instability.

To establish how tightly each component is trapped in the ITO pores, an experiment was carried out in which the cell solution was replaced during the reaction (Figure 3). An FNR@ITO/graphite electrode was made by dropcasting FNR (1 mm, 5 μL) for 5 min and subsequently rinsing thoroughly with pure water. The electrode (electroactive FNR coverage 60 pmol cm^−2^) was placed in the cell solution containing 4‐phenyl‐2‐butanol (20 mm) and NADPH (5 μm), and an oxidising potential (+0.08 V vs. SHE) was applied. After injecting ADH (final concentration 0.3 μm) the catalytic current gradually increased to reach a high steady‐state level, as expected. The electrode was then removed and stored in a vial containing the original cell solution. After thoroughly rinsing with purified water, the cell was recharged with fresh buffer containing only 4‐phenyl‐2‐butanol (20 mm). The electrode was removed from storage, rinsed thoroughly with purified water, and returned to the cell. Upon resuming measurement, the current decreased immediately, as expected, but not to zero. Instead a residual current was observed, which decreased over the course of 30 min. Readdition of NADPH produced an immediate current increase to a value that, despite the disturbance to the electrode, was about 90 % of that obtained before the experiment had been interrupted. The results confirmed that FNR and ADH are not required in solution to maintain binding on the electrode (Figure S3) and demonstrated that only NADPH is needed to restore a high rate of catalytic conversion. The results showed also that some NADP(H) is retained in the pores, and thereby undergoes many recycles before it is lost to solution. Its partial retention and concentration above the solution level was confirmed with cyclic voltammetry of NADP^+^ alone at a FNR@ITO/graphite electrode (Figure S4). Plots of current versus (scan rate)^1/2^ (corrected for the FAD signal) consistently showed a small upward deviation from linearity (standard evidence for a surface excess), even as the peaks broadened at higher scan rate.^9^

**Figure 3:**
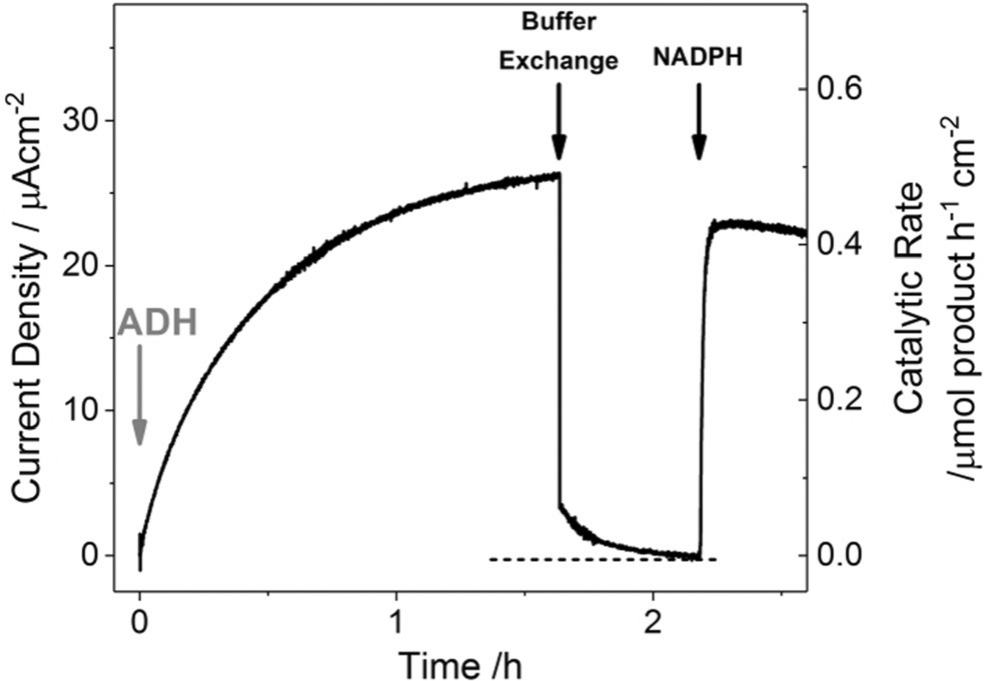
Chronoamperometric monitoring of the retention of E2 and NADPH in the ITO pores after their removal from solution. Substrate and cofactor present from the start (NADPH (5 μm), 4‐phenyl‐2‐butanol (20 mm)), ADH injected at *t*=0 to give a final concentration of 0.3 μm (see details in text). Conditions: electrode held at +0.08 V vs. SHE, 1000 rpm rotation, 20 °C, MES (50 mm), TAPS (50 mm), pH 8, cell volume 3 mL. A slow decay in residual current persists after the buffer exchange and there is an immediate rise in activity when NADPH is added.

A preformed (FNR+ADH)@ITO/glass electrode (that is, with no enzyme in solution) was used to demonstrate the reversibility and catalytic bias displayed by the (FNR+ADH) pair at pH 8.0. The quasi‐reversible cyclic voltammetry of the NADP^+^/NADPH couple transformed into reversible electrocatalysis when substrates were added (Figure 4). A ratio of 20 mm alcohol/1 mm ketone was required to equalise oxidation and reduction currents, whereas equal concentrations of alcohol and ketone (5 mm) showed no oxidation at pH 8.5 or below (Figure S5). The results confirmed not only that (FNR+ADH)@ITO is a “stand‐alone” electrode, but also that the system is strongly biased toward alcohol production—ketone being a product inhibitor.

**Figure 4:**
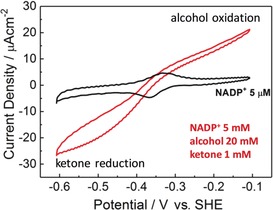
Cyclic voltammetry showing the performance of a stationary (FNR+ADH)@ITO/ITOglass electrode placed in an enzyme‐free solution for measuring electrochemical ketone/alcohol interconversion; NADP^+^ only ((—), 5 μm), 4‐phenyl‐2‐butanol and 4‐phenyl‐2‐butanone injected to give final concentrations of 20 mm and 1 mm, respectively (—). Conditions: scan rate 1 mV s^−1^, 20 °C, MES (50 mm), TAPS (50 mm), pH 8.

The experiments shown in Figure 1 were extended to three other dehydrogenases—each operating in the reducing direction. Figure 5 compares the development of catalysis observed for ADH with that obtained when comparable concentrations of RedAm, ME, and (*S*)‐IRED were introduced, all other components being present (Supporting Information) and the FNR@ITO/graphite electrode being prepared identically in each case. The rate of activity development is enzyme‐dependent; that is, RedAm≫ADH≈ME≫(*S*)‐IRED. After reaching a maximum value, the current decreased slowly for each enzyme (Figure S6). Notably, the activity that was so rapidly attained with RedAm began to decrease slowly after 30 min compared to >2 h for ADH and ME. In contrast, the activity of (*S*)‐IRED, which developed only very slowly after initiation, remained comparably constant after 6 h (Figure 5; Figure S6).

**Figure 5:**
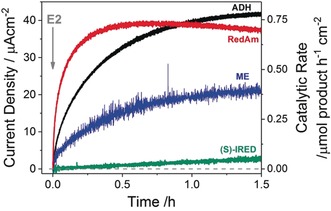
Development of catalytic activity observed as different enzymes (E2) are introduced to a FNR@ITO/graphite electrode loaded with similar amounts of FNR (as for Figure 1 D). Catalysis initiated by injecting E2 into the cell solution containing all other reagents; ADH (0.8 μm, based on native tetrameric state), ME (0.8 μm, based on monomer mass), RedAm (0.8 μm, based on native dimeric state), and (*S*)‐IRED (0.8 μm, based on native dimeric state). Substrate concentrations: ADH) 4‐phenyl‐2‐butanol (20 mm); ME) pyruvate (80 mm), MgCl_2_ (4 mm), pre‐saturated with CO_2_ and sustained; RedAm) cyclopropylamine (0.2 m), cyclohexanone (20 mm); (*S*)‐IRED) 3,4‐dihydroisoquinoline (10 mm), NADP(H) (5 μm). Electrode potential (vs. SHE) +0.08 V for oxidation and −0.44 V for reduction, 1000 rpm rotation, 20 °C. Cell volumes: ADH (3 mL), RedAm (3 mL), (*S*)‐IRED (3 mL), ME (5 mL). All currents are displayed positive to aid comparison.

The data provide a qualitative framework on which a detailed picture will eventually emerge. The results showed no obvious trend with molecular mass or oligomeric state since the two comparable and fastest adsorbing enzymes, RedAm and ADH, are dimeric and tetrameric at 62 and 172 kDa, respectively. The high affinity with which FNR (monomeric, 39 kDa) binds to ITO was evident from observations that a FNR@ITO electrode remains active for NADP^+^/NADPH cycling for several days when a second enzyme is absent.^4^ Control experiments before and after 20 h at +0.08 V or −0.44 V showed no significant changes apart from some physical cracking (Figure S7). Exposure to highly reducing conditions are known to chemically degrade ITO electrodes, but we do not expect this to occur within our range of operating potentials.^10^ The surface of ITO (isoelectric point≈6^11^) will be negatively charged at pH 8. In the chloroplast stroma, Fd and FNR form a complex in which their redox centres are aligned approximately 6 Å apart for fast electron transfer. The binding is mainly electrostatic; the negative surface of Fd interacts with a positively charged patch on FNR.^12^ A logical proposal for the electrode interaction is that FNR uses this patch to bind to the negatively charged pore walls of ITO. The isoelectric points of FNR, RedAm, and ADH are higher than ITO (Figure S8), whereas those of ME and slowly binding (*S*)‐IRED are lower.

The limiting currents in Figure 5 reflect the inherent catalytic activities of E2 and the balances between activities of FNR and E2 factored for relative coverage. At the substrate levels used, (*S*)‐IRED is the least active enzyme, as judged also by solution assays used to test the activity of each batch. Guideline (literature) values of turnover frequency *k*_cat_ and *K*_M_ are listed in Table S1 (apart from FNR, *K*_M_ values are for target substrate).

The rate of development of catalytic activity reflects the rate at which the incoming enzyme enters and binds close to the incumbent enzyme, thereby closing a local NADP(H) cycle. Certain combinations exhibit unstable catalysis. Figure 2 shows that instability is associated with the lowest preloaded level of ADH (panel A) and the highest concentration of incoming FNR (panel B), and Figure 5 shows that RedAm gives a less stable response despite binding most rapidly. These results suggest that instability may stem from aggressive displacement of the incumbent enzyme by the incoming enzyme.

The basis for the massive nanoconfinement effect is explained with a simple calculation. Within an assumed reaction volume of 10×10×10 nm^3^ (1×10^−21^ L or 1 zeptoliter) the concentration of each component of the minimal functional catalytic unit comprised of 1 FNR, 1 E2, and 1 NADP(H) would be 1.6 mm: the more mobile component, NADP(H) (*D*=4.2×10^−6^ cm^2^ s^−1^),4a requires only approximately 0.1 μs to traverse this space. Although we cannot map the 3D occupancy of pores in terms of such minimal units, it is instructive to consider each 10×10 nm face (totalling 10^12^ units cm^−2^) as an effective target area on a hypothetical flat electrode, in which case the conversion rate for ADH‐catalysed alcohol oxidation shown in Figure 5 would correspond to a steady‐state turnover frequency (per catalytic unit) in the order of 125 s^−1^. A lower density of surface units would be compensated for by the (diminishing) participation of units located deeper in the pores. “Minimal” unit is meant literally; improved catalytic rates may well require multiples of FNR or E2 (above 1:1). An alternative calculation based on participation of all the bound FNR (ca. 100 pmol cm^−2^) in the same experiment gives an empirical turnover frequency of 2 s^−1^, which is a practical value that must also represent the lower per‐unit limit.

The electrochemical nanoreactor system has important implications for technology, where cofactor regeneration is a maturing field.^13^ Optimised in terms of turnover rates, stability, and amounts/ratios of enzymes, the (FNR+E2)@ITO/support material can be scaled up and exploited as an inexpensive “plug‐in” electrode to drive, interactively, a potentially unlimited number of organic reactions depending on the identity of E2.

## Conflict of interest

The authors declare no conflict of interest.

## Supporting information

As a service to our authors and readers, this journal provides supporting information supplied by the authors. Such materials are peer reviewed and may be re‐organized for online delivery, but are not copy‐edited or typeset. Technical support issues arising from supporting information (other than missing files) should be addressed to the authors.

Supplementary
