# Supplementary material for: Electrocatalytic Volleyball: Rapid Nanoconfined Nicotinamide Cycling for Organic Synthesis in Electrode Pores
Source: Angew Chem Int Ed Engl. 2019 Feb 14;58(15):4948–52. doi: 10.1002/anie.201814370 (PMC6491978; doi:10.1002/anie.201814370)
Supplement: Supplementary file 1 — Supplementary [file ANIE-58-4948-s001.pdf]

Angewandte Chemie
Eine Zeitschrift der Gesellschaft Deutscher Chemiker

Supporting Information

Electrocatalytic Volleyball: Rapid Nanoconfined Nicotinamide Cycling for Organic Synthesis in Electrode Pores

*Clare F. Megarity[+], Bhavin Siritanaratkul[+], Rachel S. Heath, Lei Wan, Giorgio Morello, Sarah R. FitzPatrick, Rosalind L. Booth, Adam J. Sills, Alexander W. Robertson, Jamie H. Warner, Nicholas J. Turner, and Fraser A. Armstrong**

anie_201814370_sm_miscellaneous_information.pdf

Table of Contents

Table of Contents 2
Experimental Procedures 3
Materials 3
Production of FNR 3
Production of Coupled Enzymes 3
ADH, (S)-IRED and RedAm 3
ME 4
Electrode Fabrication 4
Electrochemical Measurement 4
Electron Microscopy 5
Isoelectric Focusing 5
Results and Discussion 5
Table S1. Properties of the coupled enzymes. 7
Figure S1. SEM images of the ITO pore structure. 8
Figure S2. Product detection by 1H-NMR. 9
Figure S3. Further experiments to show that E2 must bind at the electrode in order for catalysis to occur. 10
Figure S4. Scan rate dependence of FNR catalytic peaks with 20 µM $NADP^+$. 11
Figure S5. Cyclic voltammetry with equimolar concentrations of alcohol and ketone. 12
Figure S6. Extended 6 hour version of figure 5. 13
Figure S7. SEM images of the ITO electrode after holding at a potential. 14
Figure S8. Isoelectric focusing of all coupling enzymes. 15
References 16

Experimental Procedures

Materials

Indium tin oxide: Sigma; Iodine: Alfa Aesar; Isopropyl β-D-1-thiogalactopyranoside (IPTG): Fluorochem Ltd; $NADP^+$ and NADPH: Melford; (S)-(+)-4 phenyl-2-butanol: Alfa Aesar; Sodium pyruvate: Sigma Aldrich; Cyclopropylamine; Sigma Aldrich; Cyclohexanone: Sigma Aldrich; 3,4-dihydroisoquinoline: Sigma Aldrich; α-ketoglutarate: Sigma Aldrich.

Production of FNR

FNR was produced as previously described[1]. Briefly, a vector (aLICator pLATE 51) containing the gene encoding N-terminal Histagged FNR from *Chlamydomonas reinhardtii* was used to transform *Escherichia coli* cells (BL21); positive colonies were selected by resistance to ampicillin. The cells were grown aerobically for approximately 3 hours at which point they were induced by addition of IPTG to a final concentration of 1 mM and grown for a further 3 – 4 hours. The cells were then harvested by centrifugation and the pellets resuspended in cold cell resuspension buffer (50 mM Hepes; 150 mM NaCl; 10 % V/V Glycerol) and stored at – 80 °C until purification.

Purification of FNR was carried out as previously described[1]. Briefly, cells were lysed using a French press at 20 psi and centrifuged at 45000 rpm for 1 hour. The supernatant was retained and purification of FNR was carried out using a Ni^{2+} HisTrap HP affinity column (GE Healthcare Life Sciences); fractions containing FNR were selected based on the absorbance at 280 nm and 460 nm. The fractions were pooled and concentrated using Amicon® Ultra 4 mL centrifugal filters to a final volume of approximately 2 mL. The concentrated protein was passed through a desalting column (PD-10 Ge Healthcare) to remove imidazole, portioned into single use aliquots and flash frozen in liquid nitrogen before storing at -80 °C.

Production of Coupled Enzymes

ADH, (S)-IRED and RedAm

<u>Expression</u>

ADH

Under sterile conditions, the vector pET28 containing the gene for the alcohol dehydrogenase from *Thermoanerobacter ethanolicus* with mutation W110A (TesADH W110A)[2] was transformed into *E. coli* BL21 (DE3) (New England Biolabs) following the manufacturer's protocol. Colonies were cultivated on LB agar containing kanamycin at 50 µg mL^{-1}. A single colony was used to inoculate 5 mL LB media containing kanamycin at 50 µg mL^{-1} and grown overnight at 37 °C, 250 rpm. This was used to inoculate 600 mL TB autoinduction media (Formedium) containing kanamycin at 50 µg mL^{-1} in a 2 L baffled flask and left to grow for 16 h at 37 °C, 250 rpm.

(S)-IRED

The following was carried out under sterile conditions. The vector pET28 containing the gene for the (*S*)-IRED from *Streptomyces* sp GF3546 ((S)-IRED)[3] was transformed into *E. coli* BL21 (DE3) (New England Biolabs) following the manufacturer's protocol. Colonies were cultivated on LB agar containing kanamycin at 50 µg mL^{-1}. A single colony was used to inoculate 6 mL LB media containing kanamycin at 50 µg mL^{-1} and grown overnight at 37 °C, 250 rpm. This was used to inoculate 600 mL LB containing kanamycin at 50 µg mL^{-1} in a 2 L baffled flask and grown at 37 °C, to an OD_{600} of 0.6-0.8. The cells were induced with isopropyl- β-D-1-thiogalactopyranoside (IPTG) at a final concentration of 0.2 mM and grown for 18 h at 20 °C, 200 rpm.

RedAm

The following was carried out under sterile conditions. The vector pET28 containing the gene for the reductive aminase from *Aspergillus oryzae* (RedAm)[4] was transformed into *E. coli* BL21 (DE3) (New England Biolabs) following the manufacturer's protocol.

Colonies were cultivated on LB agar containing kanamycin at 50 µg mL^{-1}. A single colony was used to inoculate 6 mL LB media containing kanamycin at 50 µg mL^{-1} and grown overnight at 37 °C, 250 rpm. This was used to inoculate 600 mL LB autoinduction media (Formedium) containing kanamycin at 50 µg mL^{-1} in a 2 L baffled flask and left to grow for 72 h at 20 °C, 200 rpm.

Purification

For (*S*)-IRED, ADH and RedAm, cells were harvested by centrifugation (4000 rpm, 20 mins) and up to 5 g cell pellet was resuspended in ~ 25 mL buffer A (buffer A = 100 mM potassium phosphate, pH 7.7 + 300 mM NaCl + 20 mM imidazole). 0.1 mg mL^{-1} Lysozyme from chicken egg white (Sigma-Aldrich) was added and cells left to lyse at 30 °C, 250 rpm for 30 mins. Cells were further lysed by sonication (20 s pulse on, 20 s pulse off x 20 cycles). The suspension was centrifuged to remove insoluble components (18000 rpm, 20 mins) and the supernatant was passed through a 0.45 µm filter followed by a 0.2 µm filter. The supernatant was loaded onto a 5 mL HisTrap FF column (GE Healthcare) previously equilibrated with 10 column volumes (cv) buffer A. The column was then washed with 10 cv buffer A and the enzyme eluted with a gradient 0-100% buffer B (buffer B = 100 mM potassium phosphate, pH 7.7 + 300 mM NaCl + 1 M imidazole) over 10 cv. Fractions containing the enzyme were collected and dialysed overnight at 4 °C into 50 mM Tris buffer, pH 9 followed by concentration using a 10 kDa MWCO spin column (GE Healthcare) to >10 mg mL^{-1} and stored at -80 °C until use.

ME

Ligation-independent cloning and expression

The gene encoding malic enzyme (MaeB) was amplified by colony (*E. coli* BL21) PCR using primers based on the sequence in the database (KEGG). The amplified product was inserted into a pLATE51 vector using an aLICator ligation independent cloning kit (ThermoFisher Scientific). Upon sequence confirmation, the plasmid obtained was used to transform *E. coli* BL21 cells and a single colony was inoculated into 100 mL of LB (supplemented with ampicillin, 100 µgmL^{-1}) and grown shaking at 37 °C for 16 hours. This 100 mL subculture was diluted into 500 mL of LB (ampicillin, 100 µgmL-1) and grown shaking at 37°C for 3.5 hours. At this point, the cells were induced by the addition of IPTG to a final concentration of 0.5 mM followed by growth at 15°C for a further 20 hours.

Purification

The cells were centrifuged at 6000 rpm (4°C) for 30 minutes, resuspended in buffer (pH 7.4) containing 50 mM Hepes, 150 mM NaCl, 10% glycerol and EDTA-free protease inhibitors and stored at -80°C. Upon thawing, the cells were disrupted using a French press and centrifuged at 45000 rpm, (4°C) for 1 hour. The supernatant was loaded on a Ni^{2+} column (GE Healthcare) using Buffer A (50 mM Hepes 500 mM NaCl 50 mM Imidazole 1 mM DTT pH 7.4) and Buffer B (50 mM Hepes 500 mM NaCl 300 mM Imidazole 1 mM DTT pH 7.4). A linear (0-100%) imidazole gradient was used; the relevant fractions were selected based on malic enzyme activity tested by solution assay using a UV/Vis spectrophotometer (Perkin Elmer, Lambda 19). The protein was concentrated using a 50 kDa MWCO spin column (GE Healthcare) and dialysed overnight in 50 mM hepes 150 mM NaCl 20 mM $MnCl_2$, 1 mM DTT and 10% glycerol. Protein aliquots (20 µL) were frozen at -80°C.

Electrode Fabrication

A suspension of indium tin oxide (ITO) (0.02 g) and iodine (0.01 g) was prepared in acetone (20 mL) and sonicated for 45 minutes. Electrophoretic deposition of the ITO onto a pyrolytic graphite edge electrode (constructed in house[5]) was carried out by holding the graphite electrode approximately 1 cm from a counter electrode (ITO glass) and positioning in the ITO suspension, a potential of 10 V was then applied for 6 minutes. The ITO/graphite electrode was allowed to dry in air and before use was rinsed thoroughly in purified water (MQ water) (Millipore, 18 MΩ cm).

Electrochemical Measurement

Protein film electrochemistry (PFE) experiments in the oxidative direction (i.e. those using ADH) were performed on the bench since FNR is air stable. PFE experiments in the reduction direction (i.e. those using (S)-IRED, RedAm and ME), were performed in an

anaerobic glovebox (MBraun) with an N_2 atmosphere (O_2 < 3 ppm); this was to ensure that in these investigative experiments, there was no contribution to current from the reduction of O_2, however, when the intention is to use the FNR electrode technology for bulk syntheses, it can be used in both directions on the bench since it is air stable.

Electrochemical measurements were made with an Autolab potentiostat (Metrohm PSSTAT128N or PGSTAT101) controlled using Nova software (EcoChemie) and were conducted in a closed glass cell with a 3-electrode configuration. The reference electrode (SCE) was housed in a non-isothermal side arm connected to the bulk solution in the main compartment by a Luggin capillary, the counter electrode (Pt) and working ITO@graphite electrode were placed in the main compartment. The main compartment was water jacketed to control temperature. The working electrode was controlled via a rotator so that mass transport limitation was overcome, by rotation at 1000 rpm. The reference potential was converted to the standard hydrogen electrode (SHE) scale using $E_{SHE} = E_{SCE}$ + 0.241 V at 25 °C [6].

The FNR@ITO/graphite electrode was prepared by dropping a small aliquot of FNR (3-5 µL) onto the ITO surface and incubating at room temperature for 5 min before rinsing thoroughly in a stream of MQ water and immersing in the cell solution. For experiments which involved the addition of coupling enzyme to the electrode, ADH was dropped onto the ITO layer and incubated at room temperature for varying lengths of time before rinsing to remove excess un-adsorbed enzyme, and immersing in the cell solution.

The second enzyme (either coupling enzyme or FNR in the case where ADH was adsorbed to the electrode first) was injected into the cell solution containing the working FNR@ITO electrode using a needle which had been pre-positioned into the solution. Small volumes were involved therefore after injection the needle and the syringe were flushed by injecting some of the cell solution up and down at least 5- 10 times.

Electron Microscopy

Scanning electron microscopy (SEM) was performed on a Zeiss NVision 40 FIB-SEM, using both secondary electron Evarhart-Thornley and in-lens detectors at an accelerating voltage of 5 kV.

Transmission electron microscopy (TEM) images were acquired on a JEOL 3000F at an accelerating voltage of 300kV. The sample was prepared by dry rubbing transfer of the thin film to a holey carbon grid

Isoelectric Focusing

Isoelectric focusing was carried out as described in the manufacturer's instructions (Novex IEF gels, ThermoFisher Scientific). Briefly, the pre-cast isoelectric gel (Novex™ pH 3-10 IEF) was inserted into the electrophoresis tank and pre-chilled (4 °C) cathode buffer (Novex) was poured into the upper chamber and left for approximately 10 min to ensure there were no leaks into the lower chamber. Pre-chilled (4 °C) anode buffer (Novex) was then poured into the lower chamber. The tank was then immersed in a water bath which was topped up with ice throughout the electrophoresis to prevent overheating of the cathode and anode buffers and gel.

Aliquots (10 µL) of each enzyme were prepared to a final concentration of 1.2 $mgmL^{-1}$ (in sample buffer (Novex)). The samples were kept on ice until loading into the wells of the gel.

The samples were electrophoresed for 1 hour at 100 V followed by 1 hour at 200 V and finally 30 mins at 500 V. The gel was fixed by immersion in 12 % trichloroacetic acid and subsequently stained in Instant Blue (Expedeon).

Results and Discussion

Figure S3 shows preliminary experiments which were initially carried out to confirm that only the enzyme molecules adsorbed in pores contribute to current. Buffer exchange experiments similar to that shown in Figure 3 were carried out using ADH, RedAm, (S)-IRED and glutamate dehydrogenase (GDH) (our initial exemplar coupling enzyme used at the outset of work on this technology[1]). FNR was applied to an ITO@graphite electrode and incubated at room temperature for 5 minutes before rinsing and placing in the electrochemical cell solution. Coupling to ADH and RedAm was monitored by chronoamperometry (Figure S3 A and B). Coupling to

GDH and (S)-IRED was monitored using cyclic voltammetry (Figure S3 D and E). In each experiment, nicotinamide cofactor and all substrates were present before the addition of coupling enzyme, upon which, an immediate increase in rate is observed. In chronoamperometry this is observed as an increase in current with time and in cyclic voltammetry this is observed as increasing sigmoidal reduction waves. In figure S3 A and B, the rate enhancement continued to a maximum at which time the measurement was paused, the FNR@ITO/graphite electrode was removed and stored in buffer and the cell solution was stored to one side while the cell was rinsed thoroughly with MQ H_2O and then fresh buffer. The clean cell was refilled with fresh buffer containing all substrates to the same concentrations as before and importantly, no coupling enzyme. Upon resuming measurement, it was observed that the maximum rate was maintained. This proves that the coupling enzyme molecules which contribute to rate are indeed confined within the electrode pores along with FNR and that any un-adsorbed enzyme in solution is redundant. Moreover, having confirmed this we also concluded that the immediate growth in current upon injection of enzyme to the solution reflects the adsorption of enzyme molecules into the ITO electrode pores. Figure S3 C and D show the results of a similar experiment monitored by cyclic voltammetry instead of chronoamperometry. Again after injection of the coupling enzyme, increasing sigmoidal waves are observed. At maximum activity (taken to be when the voltammograms overlay) the electrode and cell solution were removed and stored to one side while the cell was cleaned thoroughly and fresh buffer solution containing all substrates to the original concentrations replaced. Again, the current does not decrease.

An additional experiment in which an FNR@ITO/Ti electrode was contained within a tightly fitted dialysis membrane (which allowed small substrate molecules through but which prevented large enzyme molecules through) also indicated that the coupling enzyme molecules in solution did not contribute to the rate (Figure S3 E). The membrane prevented downstream enzyme molecules' access to the ITO layer. Midway through the experiment the membrane was teared to allow access and immediately an increase in current was observed.

Table S1. Properties of the coupled enzymes.

The reaction catalysed is shown in the direction used (large arrow). All parameters taken from literature apart from the isoelectric point which was experimentally determined (Fig. S8) and the FNR coverage measured at the end of each chronoamperometry experiment; the longest dimension of each enzyme was estimated using PyMol.

Enzyme	Oligomeric State	Molecular Weight /kda	Longest Dimension/Å	Isoelectric Point	FNR coverage at end of expt. in Fig. 5 /pmolcm^{-2}	*k*cat/ s^{-1}	K_m/mM	*k*cat/K_m /mM^{-1}s^{-1}	PDB
**FNR**	Monomer	39	60	6 < FNR < 6.9	Not Applicable	174[7] (pH 8.0)	0.033[7] (pH 8.0)	5273	1GJR[8]
**ADH**	Tetramer	172	90	7.4 < ADH > 7.8	75	31.1[9] (pH 8.9)	6.3[9] (pH 8.9)	4.94	2NVB[10]
**ME**	Octamer	664	Structure not solved	5<ME<6	105	0.38[11] (pH 7.0)	6.2[11] (pH 7.0)	0.06	Not solved
**RedAm**	Dimer	62	88	6 < RedAm < 6.9	46	Not Available	Not Available	Not Available	5G6R[4]
**(S)-IRED**	Dimer	62	88	~ 6	103	0.445[3] (pH 7.0)	0.63[3] (pH 7.0)	0.71	4OQY[12]

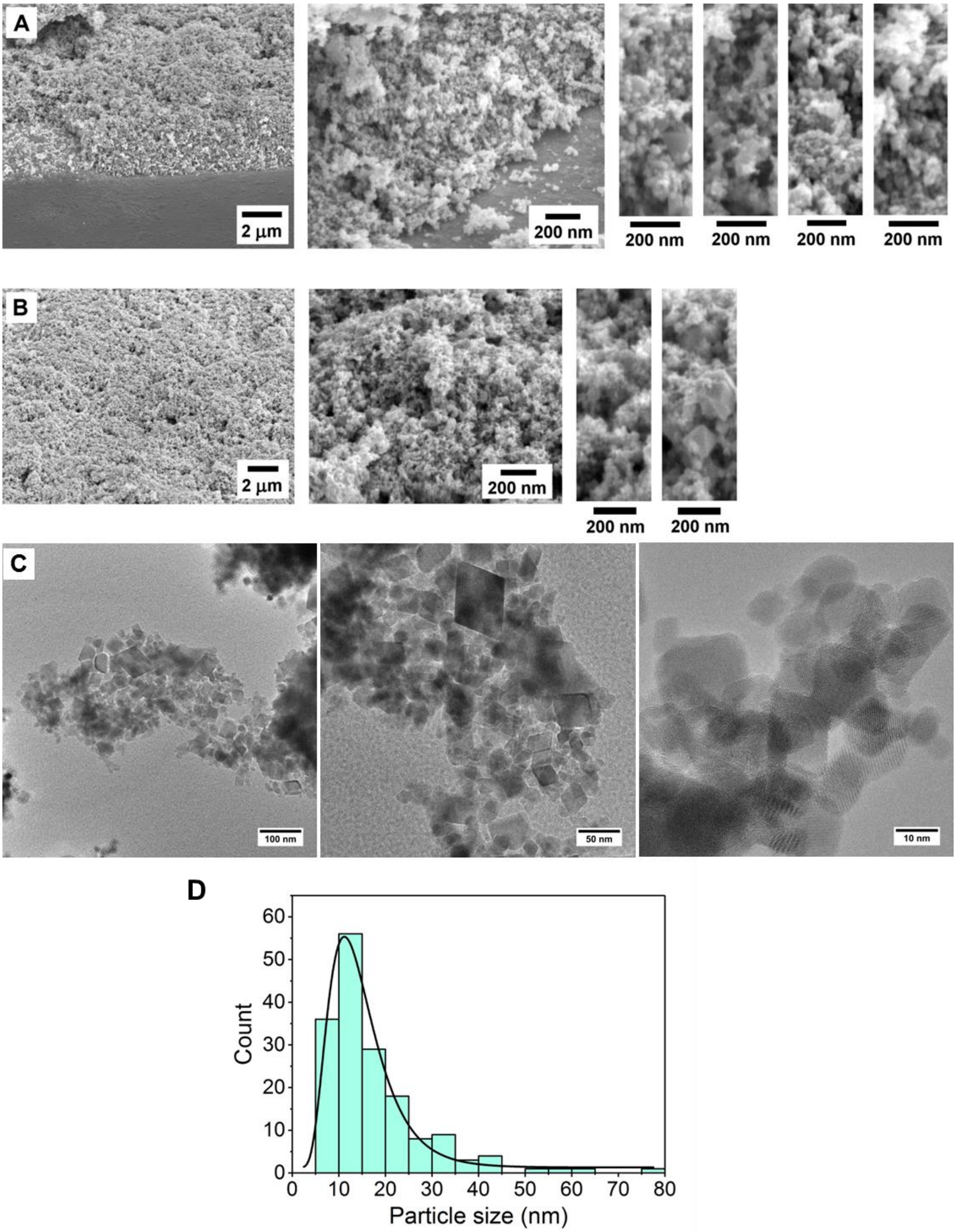

Figure S1. SEM images of the ITO pore structure.

A: SEM images from a cross-section of the ITO film. **B:** Surface as-deposited (i.e., top-down) SEM images of the ITO. **C:** TEM images of transferred ITO particles. **D:** Particle size distribution from TEM and a log-normal fit.

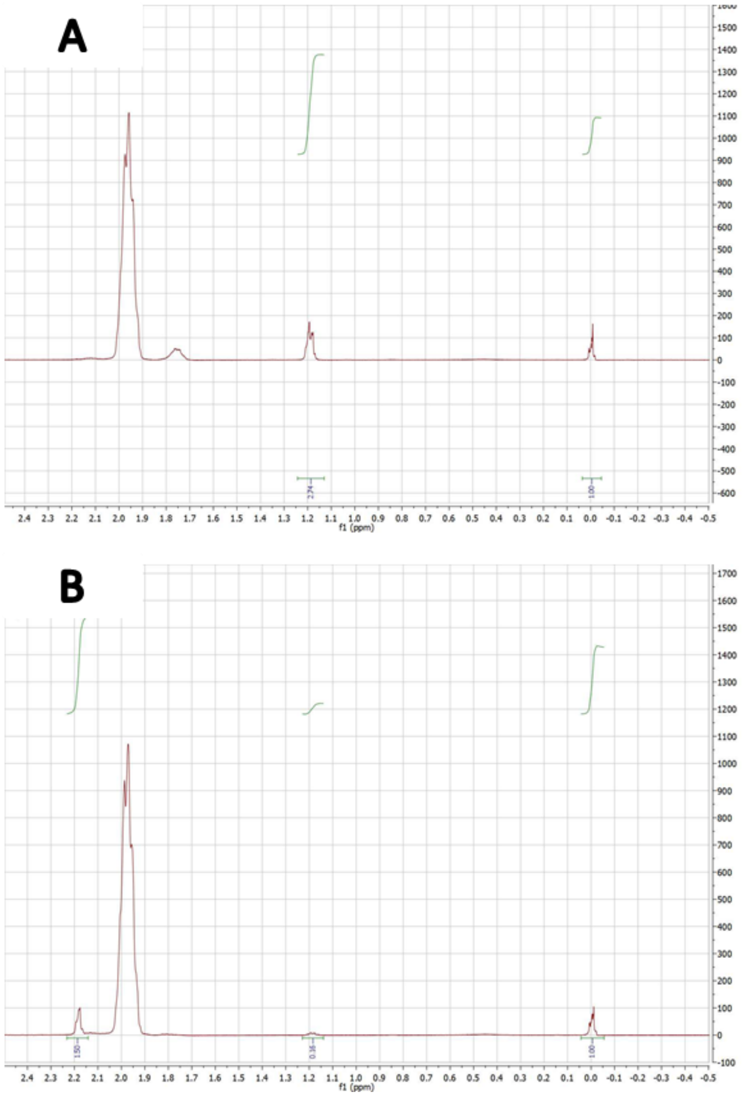

Figure S2. Product detection by 1H-NMR.

A: Spectrum for (S)-4-phenyl-2-butanol (10.5 mM). **B:** Spectrum obtained at the end of bulk electrolysis for the oxidation of (S)-4-phenyl-2-butanol using (FNR+ADH)@ITO/Ti foil, electrode area = 7.2 cm^2. In this experiment NADP$^+$ regeneration by FNR was driven by O_2 reduction at a platinum electrode. Peak assignment: (S)-4-phenyl-2-butanol: δ 1.18 (m, 3H); 4-phenyl-2-butanone: δ 2.18 (m, 3H); internal standard 3-(Trimethylsilyl)propionic-2,2,3,3-d4 acid sodium salt (TMSP): δ 0; TAPS buffer: ca. δ 1.95.

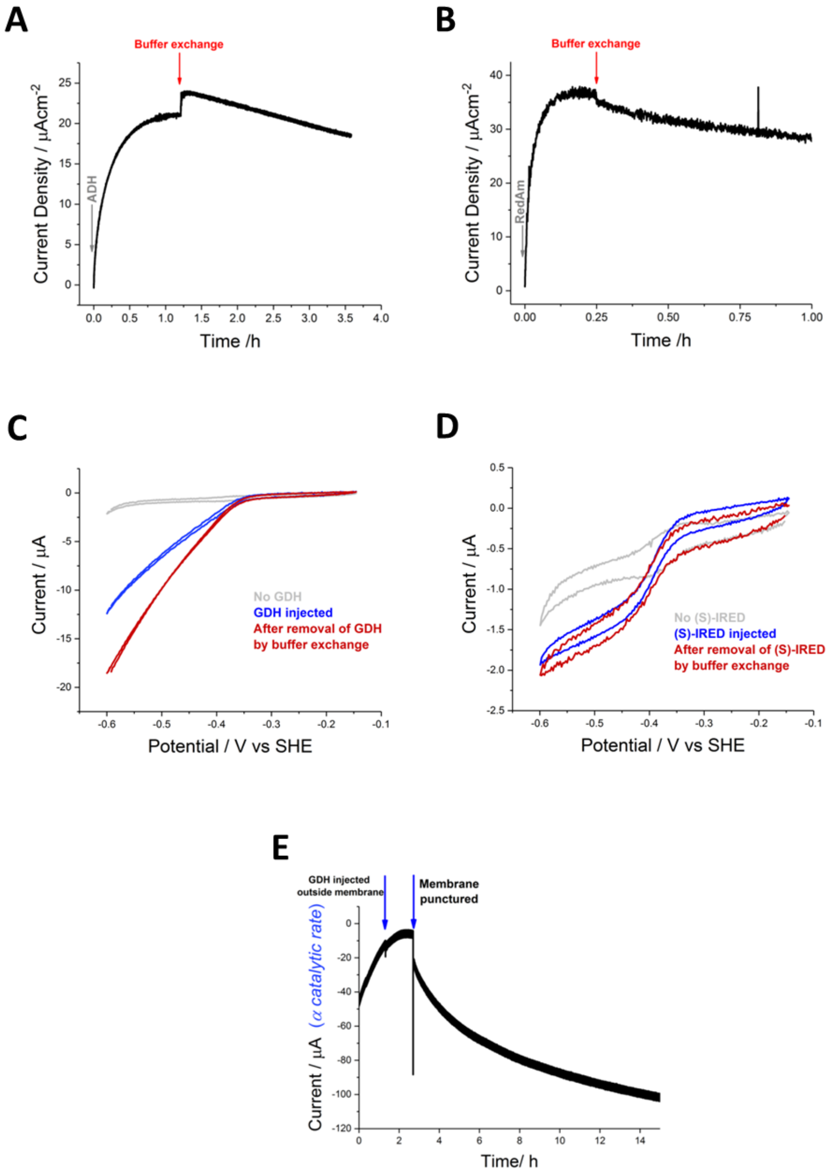

Figure S3. Further experiments to show that E2 must bind at the electrode in order for catalysis to occur.
A: Chronoamperometry to monitor coupling of FNR@ITO/graphite to alcohol oxidation by ADH. $NADP^+$ and (S)-(+)-4 phenyl-2-butanol were present from the start at 5 µM and 20 mM respectively before the addition of ADH (0.3 µM (0.05 $mgml^{-1}$)) (grey arrow); buffer exchange to remove all traces of ADH (red arrow). **B:** Chronoamperometry to monitor coupling to reductive amination by RedAm; $NADP^+$, cyclohexanone and cyclopropylamine at 5 µM, 20 mM and 200 mM respectively were all present before the addition of RedAm (0.8 µM (0.05 $mgml^{-1}$)) (grey arrow); buffer exchange (red arrow). Experiment conditions: electrode rotated at 1000 rpm; 20 °C; 50 mM MES, 50 mM TAPS, pH 8. **C:** Cyclic voltammetry to monitor coupling to the reduction of α-ketoglutarate by GDH (0.2 µM (0.05 $mgml^{-1}$) (blue scan); $NADP^+$, α-ketoglutarate and NH_4^+ present from the start at 5 µM, 50 mM, and 60 mM respectively; scan after buffer exchange shown in red. **D:** Cyclic voltammetry to monitoring coupling to imine reduction by (S)-IRED (0.8 µM (0.05 $mgml^{-1}$) (blue scan). $NADP^+$, 3,4-dihydroisoquinoline, 5 µM and 10 mM respectively (1% DMSO). Experiment conditions for A-D: electrode rotated at 1000 rpm in chronoamperometry and stationary in cyclic voltammetry; 20 °C; 50 mM MES, 50 mM TAPS, pH 8. **E:** Prevention of GDH adsorption into the ITO layer by a tight fitting dialysis membrane. The FNR@ITO/Ti was encased in a tight membrane and the current monitored. GDH was injected into the bulk solution but no coupled activity was observed. The membrane was teared as indicated, after which, an increase in current was immediately observed as the ITO layer was now accessible to the GDH molecules. Experiment conditions: 50 mM MES 50 mM TAPS pH 8; 20 °C; bulk solution stirred throughout with a magnetic sir bar; GDH injected to a final concentration of 0.2 µM (0.05 $mgml^{-1}$); $NADP^+$ 20 µM ; α-ketoglutarate 20 mM ; NH_4^+ 60 mM; cell volume: **A:** 3 mL; **B:** 5 mL; **C:** 3 mL; **D:** 3 mL; **E:** 45 mL.

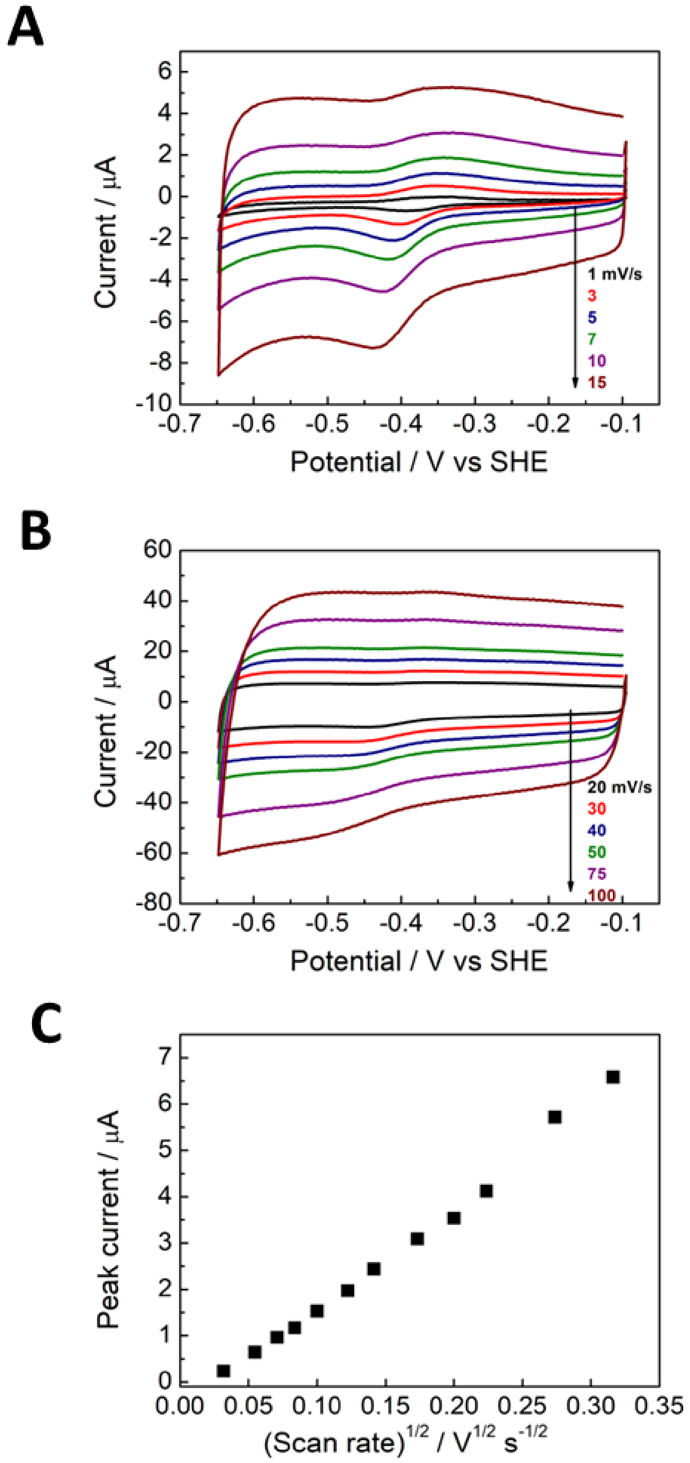

Figure S4. Scan rate dependence of FNR catalytic peaks with 20 µM $NADP^+$.

A: Cyclic voltammograms showing the change in catalytic peaks at scan rates ranging from 1 to 15 mVs^{-1}. **B:** Cyclic voltammograms showing the change in catalytic peaks at scan rates ranging from 20 – 100 mVs^{-1}. **C:** Peak reductive current vs scan rate$^{1/2}$. The peak reductive current was corrected for contribution from the FNR non-turnover peak by subtracting the non-turnover signal at the same potential and scan rate as that for the catalytic peak. Experiment conditions 20 °C, 20 µM $NADP^+$, 50 mM MES 50 mM TAPS pH 8, stationary electrode; cell volume: 3 mL.

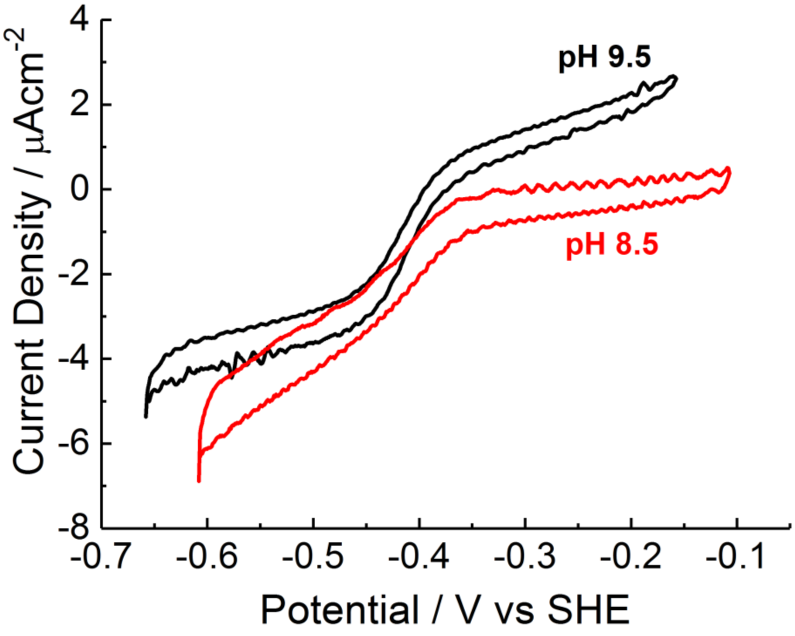

Figure S5. Cyclic voltammetry with equimolar concentrations of alcohol and ketone.

Cyclic voltammetry to show the bias of a stationary (FNR + ADH)@ITO/ITOglass electrode placed in an enzyme-free solution containing equimolar amounts of (s)-(+)-4-phenyl-2-butanol and 4-phenyl-2-butanone (5 mM). The catalytic activity was first measured at pH 9.5 (black trace); the same electrode was removed and rinsed before measurement at pH 8.5 (red trace). Experiment conditions: Scan rate 1 mVs^{-1}; 20 °C; 50 mM TAPS 50 mM CHES; stationary electrode; cell volume: 2.5 mL.

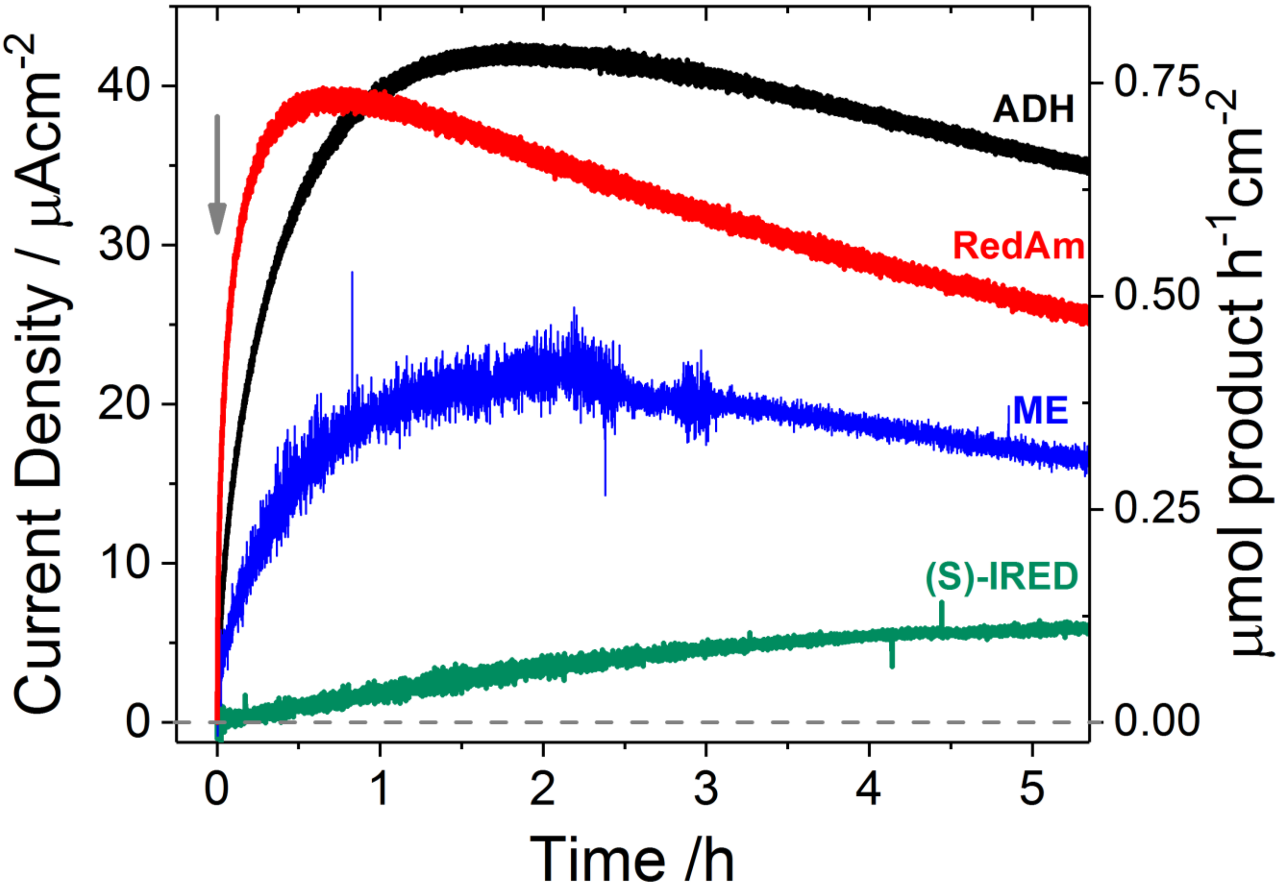

Figure S6. Extended 6 hour version of figure 5.

Live monitoring of the development of catalytic activity observed as different enzymes are introduced to a FNR@ITO/graphite electrode, the amount of FNR being approximately equal in each experiment. Incoming enzymes are: ADH (black), (S)-IRED (green), RedAm (red) and ME (blue). All substrates were present from the start and the coupled reaction was initiated by the injection of enzyme into the cell solution (grey arrow). Final enzyme concentrations: ADH: 0.8 µM (based on its native tetrameric state) ; ME 0.8 µM (based on the monomer molecular weight); RedAm 0.8 µM (based on its native dimeric state); (S)-IRED 0.8 µM (based on its native dimeric state). Substrate concentrations: ADH: (S)-(+)-4 phenyl-2-butanol 20 mM; ME: Sodium pyruvate 80 mM; $MgCl_2$ 4 mM; buffer solution pre-saturated with CO_2 and CO_2 flow maintained in the headspace throughout the experiment; RedAm: cyclopropylamine 200 mM; cyclohexanone 20 mM (0.7% V/V DMSO); (S)-IRED: 3,4-dihydroisoquinoline 10 mM (1% V/V DMSO); $NADP^+$ 5 µM for each experiment. For experiments run in the oxidation direction, the potential was held at 0.08 V vs SHE and for reduction, the potential was held at -0.44 V vs SHE. cell volume: ADH, RedAm and (S)-IRED: 3 mL; ME: 5mL. All currents are displayed positive to aid comparison.

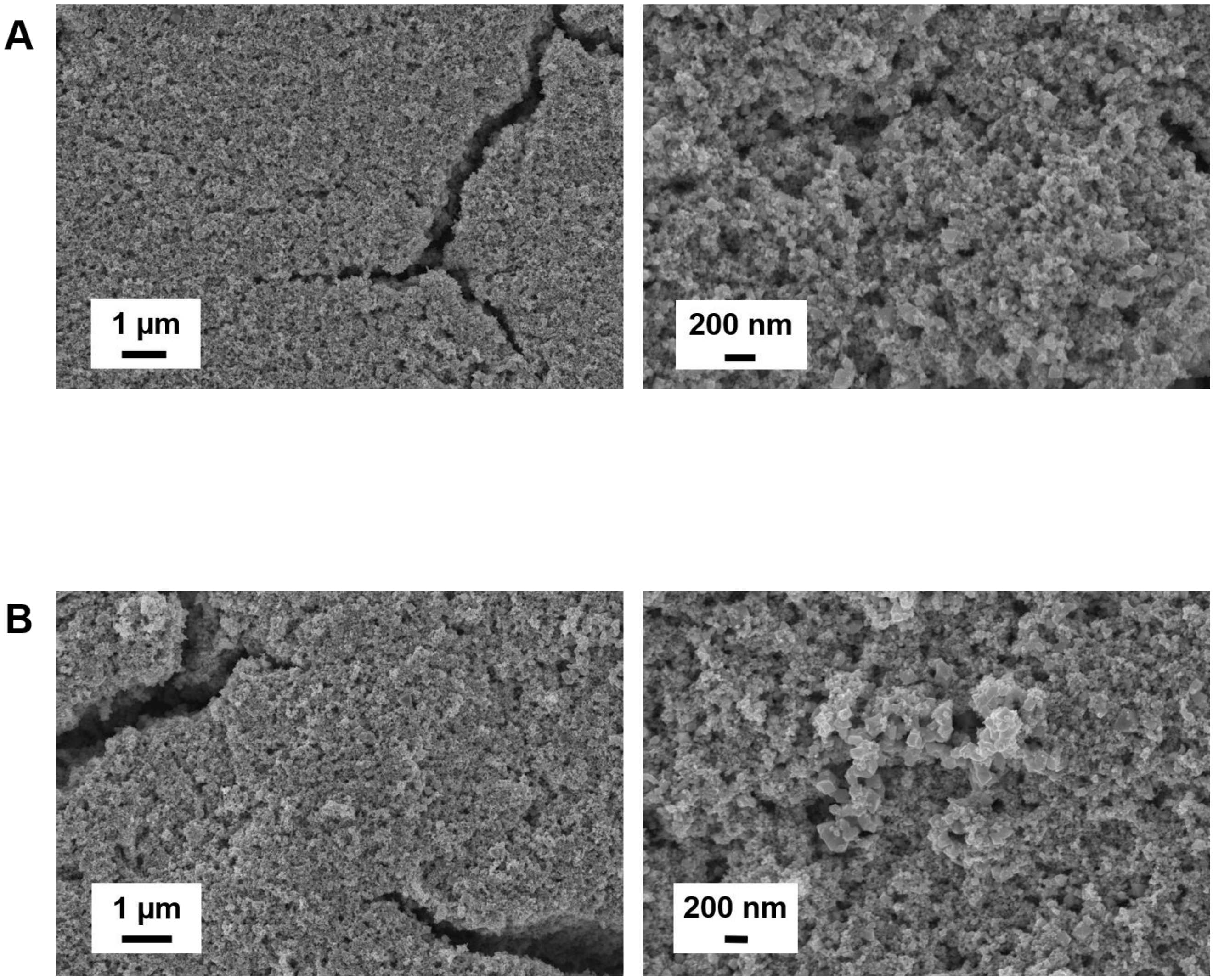

Figure S7. SEM images of the ITO electrode after holding at a potential.

SEM images of an ITO electrode after holding for 20 h at **A:** -0.44 V vs SHE and **B:** +0.08 V vs SHE.

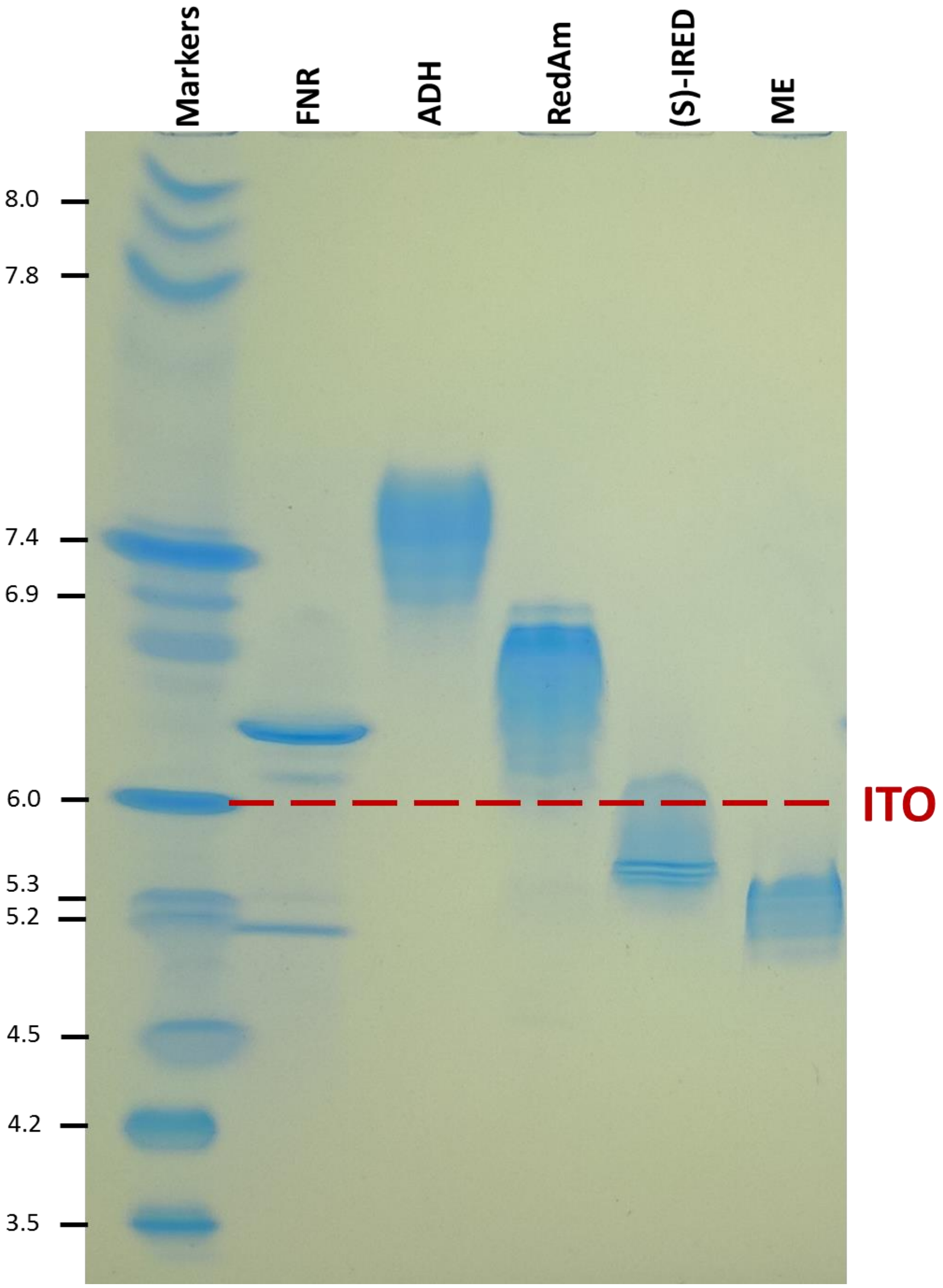

Figure S8. Isoelectric focusing of all coupling enzymes.

Gel pH gradient: 3 -10; markers: IEF markers 3-10, SERVA Liquid mix. 8 µg enzyme loaded in each well. Samples electrophoresed for 1 hour at 100 V followed by 1 hour at 200 V and final 30 mins at 500 V. Gel fixed by immersion in 12% trichloroacetic acid and stained in Instant Blue.

References

[1] B. Siritanaratkul, C. F. Megarity, T. G. Roberts, T. O. M. Samuels, M. Winkler, J. H. Warner, T. Happe, F. A. Armstrong, *Chemical Science* **2017**, *8*, 4579-4586.
[2] S. L. Montgomery, J. Mangas-Sanchez, M. P. Thompson, G. A. Aleku, B. Dominguez, N. J. Turner, *Angewandte Chemie International Edition* **2017**, *56*, 10491-10494.
[3] F. Leipold, S. Hussain, D. Ghislieri, N. J. Turner, *ChemCatChem* **2013**, *5*, 3505-3508.
[4] G. A. Aleku, S. P. France, H. Man, J. Mangas-Sanchez, S. L. Montgomery, M. Sharma, F. Leipold, S. Hussain, G. Grogan, N. J. Turner, *Nature Chemistry* **2017**, *9*, 961.
[5] A. Sucheta, R. Cammack, J. Weiner, F. A. Armstrong, *Biochemistry* **1993**, *32*, 5455-5465.
[6] B. a. Faulkner, *Electrochemical methods : fundamentals and applications*, 2 ed., Wiley, Chichester, **1980**.
[7] P. Decottignies, V. Flesch, C. Gérard-Hirne, P. Le Maréchal, *Plant Physiology and Biochemistry* **2003**, *41*, 637-642.
[8] J. A. Hermoso, T. Mayoral, M. Faro, C. Gomez-Moreno, J. Sanz-Aparicio, M. Medina, *Journal of molecular biology* **2002**, *319*, 1133-1142.
[9] J. M. Patel, M. M. Musa, L. Rodriguez, D. A. Sutton, V. V. Popik, R. S. Phillips, *Organic & Biomolecular Chemistry* **2014**, *12*, 5905-5910.
[10] E. Goihberg, O. Dym, S. Tel-Or, L. Shimon, F. Frolow, M. Peretz, Y. Burstein, *Proteins* **2008**, *72*, 711-719.
[11] F. P. Bologna, C. S. Andreo, M. F. Drincovich, *Journal of bacteriology* **2007**, *189*, 5937-5946.
[12] T. Huber, L. Schneider, A. Präg, S. Gerhardt, O. Einsle, M. Müller, *ChemCatChem* **2014**, *6*, 2248-2252.
